# Supplementary material for: Fish predation hinders the success of coral restoration efforts using fragmented massive corals
Source: PeerJ. 2020 Oct 2;8:e9978. doi: 10.7717/peerj.9978 (PMC7534677; doi:10.7717/peerj.9978)
Supplement: Supplemental Information 1 — Shown are the coefficient estimates in relation to a reference point for each factor, standard error of estimates, t statistics, and p values for the null hypothesis of no difference with respect to the reference point. Significant coefficients are bolded. Null deviance, deviance, and D-squared present a quality-of-fit of the model. [file peerj-08-9978-s001.docx]

| Model Response | Coefficient | Estimate | Std.Error | t-statistic | p-value | Null Deviance | Deviance | D-squared |
| --- | --- | --- | --- | --- | --- | --- | --- | --- |
| **Proportion of corals removed** | Intercept | -2.44 | 0.35 | -7.02 | **2.31E-12** | 87.77 | 29.40 | 0.67 |
|  | *O. faveolata* | 0.45 | 0.37 | 1.22 | 2.23E-01 |  |  |  |
|  | *P. clivosa* | 1.38 | 0.36 | 3.82 | **1.33E-04** |  |  |  |
|  | *P. strigosa* | 1.70 | 0.39 | 4.38 | **1.20E-05** |  |  |  |
|  | Reef 2 | 0.41 | 0.21 | 1.94 | 5.19E-02 |  |  |  |
|  | Reef 3 | -0.93 | 0.41 | -2.30 | **2.14E-02** |  |  |  |
|  | Removal @ 6 mo | 0.45 | 0.20 | 2.26 | **2.36E-02** |  |  |  |
