# Supplementary material for: Fish predation hinders the success of coral restoration efforts using fragmented massive corals
Source: PeerJ. 2020 Oct 2;8:e9978. doi: 10.7717/peerj.9978 (PMC7534677; doi:10.7717/peerj.9978)
Supplement: Supplemental Information 2 — Shown are estimates, standard errors (SE), Z statistics, and P values. Significant comparisons are bolded. Mcav = Montastraea cavernosa, Ofav = Orbicella faveolata, Pcliv = Pseudodiploria clivosa, Pstri = Pseudodiploria strigosa. [file peerj-08-9978-s002.docx]

| Model Response | Pairwise comparisons | Estimate | SE | Z | p-value |
| --- | --- | --- | --- | --- | --- |
| **Proportion of corals removed by predation** | *Ofav - Mcav* | 0.45 | 0.37 | 1.22 | 8.23E-01 |
|  | *Pcliv - Mcav* | 1.38 | 0.36 | 3.82 | **1.25E-03** |
|  | *Pstri - Mcav* | 1.70 | 0.39 | 4.38 | **1.00E-03** |
|  | *Pcliv - Ofav* | 0.93 | 0.24 | 3.87 | **1.00E-03** |
|  | *Pstri - Ofav* | 1.25 | 0.28 | 4.40 | **1.00E-03** |
|  | *Pstri - Pcliv* | 0.32 | 0.28 | 1.17 | 8.51E-01 |
|  | Reef 2 - Reef 1 | 0.41 | 0.21 | 1.94 | 3.33E-01 |
|  | Reef 3 - Reef 1 | -0.93 | 0.41 | -2.30 | 1.59E-01 |
|  | Reef 3 - Reef 2 | -1.35 | 0.41 | -3.31 | **8.53E-03** |
|  | 1 week - 6 mo. | 0.45 | 0.20 | 2.26 | **1.73E-01** |
