# Supplementary material for: Fish predation hinders the success of coral restoration efforts using fragmented massive corals
Source: PeerJ. 2020 Oct 2;8:e9978. doi: 10.7717/peerj.9978 (PMC7534677; doi:10.7717/peerj.9978)
Supplement: Supplemental Information 3 — Includes scientific and common names as well as average fish abundance with standard deviation for all three outplant sites. [file peerj-08-9978-s003.docx]

| **Scientific Name** | **Common Name** | **Average Abundance per survey**  **at Reef 1 (individuals m^-2^) ± SD** | **Average Abundance per survey**  **at Reef 2 (individuals m^-2^) ± SD** | **Average Abundance per survey**  **at Reef 3 (individuals m^-2^) ± SD** |
| --- | --- | --- | --- | --- |
| *Chaetodon striatus* | Banded Butterflyfish | 0.8 ± 1.1 | 0.2 ± 0.6 | 0.4 ± 1.1 |
| *Chaetodon capistratus* | Foureye Butterflyfish | 1.1 ± 1.5 | 1.8 ± 1.2 | 0 ± 0 |
| *Chaetodon sedentarius* | Reef Butterflyfish | 0 ± 0 | 0 ± 0 | 0.2 ± 0.6 |
| *Chaetodon ocellatus* | Spotfin Butterflyfish | 1.1 ± 1.9 | 0 ± 0 | 0.1 ± 0.4 |
| *Stegastes partitus* | Bicolor Damselfish | 13.9 ± 6.4 | 11 ± 3.9 | 1.4 ± 2.2 |
| *Stegastes variabilis* | Cocoa Damselfish | 0 ± 0 | 0 ± 0 | 0.1 ± 0.3 |
| *Stegastes fuscus* | Dusky Damselfish | 1.6 ± 2.8 | 1.2 ± 1.3 | 0 ± 0 |
| *Scarus coelestinus* | Midnight Parrotfish | 0.1 ± 0.3 | 0 ± 0 | 0 ± 0 |
| *Scarus taeniopterus* | Princess Parrotfish | 0.8 ± 1.1 | 0.7 ± 1.6 | 0 ± 0 |
| *Scarus vetula* | Queen Parrotfish | 0.1 ± 0.3 | 1.0 ± 1.9 | 0 ± 0 |
| *Scarus guacamaia* | Rainbow Parrotfish | 0.1 ± 0.3 | 0 ± 0 | 0 ± 0 |
| *Sparisoma aurofrenatum* | Redband Parrotfish | 3.8 ± 4.0 | 1.5 ± 1.1 | 1.4 ± 2.5 |
| *Sparisoma chrysopterum* | Redtail Parrotfish | 0.3 ± 0.8 | 2.4 ± 2.2 | 0.4 ± 0.9 |
| *Sparisoma viride* | Stoplight Parrotfish | 2.4 ± 3.6 | 4.0 ± 3.3 | 0.4 ± 0.8 |
| *Scarus iseri* | Striped Parrotfish | 2.2 ± 2.6 | 2.8 ± 3.0 | 1.4 ± 2.1 |
| *Sparisoma rubripinne* | Yellowtail Parrotfish | 4.4 ± 13.2 | 0.9 ± 1.3 | 0.9 ± 2.2 |
| *Acanthurus coeruleus* | Blue Tang | 3.7 ± 3.6 | 4.3 ± 2.2 | 0 ± 0 |
| *Acanthurus chirurgus* | Doctorfish | 3.3 ± 3.8 | 5.1 ± 5.0 | 1.7 ± 1.9 |
| *Acanthurus tractus* | Ocean Surgeonfish | 7.7 ± 6.5 | 3.5 ± 4.7 | 2.1 ± 3.1 |
| *Balistes capriscus* | Gray Triggerfish | 0 ± 0 | 0 ± 0 | 0.3 ± 0.5 |
| *Balistes vetula* | Queen Triggerfish | 0.1 ± 0.5 | 0 ± 0 | 0.2 ± 0.4 |
| *Halichoeres bivittatus* | Slippery Dick | 13.1 ± 7.5 | 9.8 ± 9.3 | 7.4 ± 3.5 |
| *Halichoeres garnoti* | Yellowheaded Wrasse | 8.6 ± 8.5 | 14.8 ± 12.8 | 5.9 ± 5.7 |
| *Halichoeres maculipinna* | Clown Wrasse | 0 ± 0 | 0 ± 0 | 0.6 ± 1.4 |
| *Thalassoma bifasciatum* | Blueheaded Wrasse | 17.5 ± 11.6 | 4.6 ± 7.6 | 3.1 ± 4.1 |
